# Round the Hospitals

**Published:** 1916-06-03

**Authors:** 


### ROUND THE HOSPITALS.

Nothing has yet been finally settled fixing the qualifications and regulations under which nurses will be accepted for the Register of the College of Nursing. This matter is, however, being carefully considered by the Council, which will probably formulate and approve regulations at their next meeting. The Hon. A. Stanley, chairman, hopes to be in a position shortly to summon a meeting of those who have been nominated by hospitals, Poor-Law infirmaries, and others, which will then decide upon the members to be elected on the consultative board.

---

The importance of dietetics cannot be questioned. They are not yet sufficiently studied or recognised in England. It is far different in the United States. Yet the recovery and restoration of many injured men might be expedited by a system which provides for a continuous inspection of the cooking and food arrangements of our military hospitals and military camps, and of all Red Cross and Territorial hospitals too. The diet is frequently rough, the cooking, gravely bad and unsatisfactory at first, has improved of late. But even so, it is still marred often by deficient organisation, by bad carving, and the faulty distribution of the food to the men. Further, although the ordering and supply of food may be good, the ordering and arrangement as represented by each day's menu show an absence of knowledge of dietetics and food values which is lamentable and even discreditable to those responsible. Some of these points are clearly indicated in a letter which will be found on p. 226, to which we direct the attention of the Commandant and matron of every hospital where wounded are received. We shall be glad if some of these officers will be good enough to send us any experience and difficulties encountered, that we may help them to a speedy remedy where needed.

---

As the war progresses the importance of diet in military hospitals and military convalescent camps, and indeed in all institutions where wounded or sick warriors are received, becomes forcibly apparent. For the first twelve or eighteen months, even in military camps, the diet and food arrangements were not always satisfactory. An ably conducted hospital like the Royal Connaught Hospital, Aldershot, acquired a rather bad name because cases requiring hospital care were evacuated to a military camp where hospital care was not provided. An explanation from the Commandant of how these cases have occurred would be valuable, and Surgeon-General Keogh might look into the matter. The practice has proved cruel to the patient, unjust to the camp authorities, and dangerous to the Army, in the sense that men who might otherwise be made fit to rejoin the Colours may by such premature evacuation become chronically incapacitated. Dietetically these cases often require special treatment, and, apart from surgical disabilities, a camp is not a suitable place where they can obtain it. Further, the avoidable expense, the dangerous delays, the hardship to the fighting men, and the loss of valuable members of the fighting force which premature evacuation may entail call for action to render any further exhibitions of similar incapacity practically impossible.

There is a picture in the Academy, entitled "Under the Red Cross," which gives a curiously unreal picture of how trained nurses attend to wounded soldiers. The conditions depicted are precisely how one would expect a wounded man to be dressed by untrained women, but this man is being bandaged by an elderly lady abundantly adorned with medals. She is wearing a British nursing sister's uniform, and has evidently not washed her hands, for her cuffs are fastened closely at the wrist. The patient, who appears to have a wound in the shoulder and another in the knee, is lying on a low couch, a sheet is under him and another has almost slipped off him. The whole of his body, which should of course be covered with blankets, is exposed. His uniform is thrown under the couch, and two nurses, apparently Army sisters, who have thrown off their capes, are helping the patient to become moribund. One is kneeling on the floor in order to remove the puttee from the wounded leg, a thing even an untrained nurse would hesitate to do unless her stock of clean aprons was inexhaustible; the other is helping to bandage the shoulder. One basin only is to be seen, and that a very small one. Such a picture should not have escaped criticism by someone before it took its place on the walls of the Academy. Why not add an expert to the Hanging Committee during war time?

The birthday of Florence Nightingale has called forth an interesting letter in our contemporary the *Common Cause*. Miss Charlotte Stopes describes in it the fact that a letter exists in the Record Office written to an officer by Miss Nightingale and sent by him to his superior officer with the following comment: "I think that it is time that we crushed the pretensions of Miss Nightingale to unlimited and almost irresponsible command over her nurses attached to the Army in the East." In these days, when the value of Miss Nightingale's work is abundantly established, it is well to be reminded of the bitter opposition which she had to encounter. Miss Nightingale was too strong to be crushed, but the battle she fought is not yet won, and matrons are to-day fighting the same foes under much the same conditions as sixty years ago, and we know that in Mesopotamia the state of affairs has excelled in its horrors anything experienced by our soldiers in the Crimea.

Another letter, written by Miss Alicia Leith, tells of a mistake she made which called forth a charming little note from "the Lady of the Lamp." Having erroneously learnt that May 15 was Miss Nightingale's birthday, Miss Leith sent a birthday book which she had published to Miss Nightingale for her signature, having specially chosen the following lines for that date:

> Helper of the poor and suffering,
> Victor in a noble strife,
> Singer of a noble poem,
> Such the honours of my life.

The book was returned with Miss Nightingale's signature opposite the lines for May 12, which ran as follows:

> Nor noise nor blaze attend my peaceful path;
> Nor were it otherwise, should I desire
> That noise and blaze of mine won any heart.
> Henry Taylor.

The following note was enclosed:

Dear Madam,—As you ask me, I have written my name opposite May 12th, my "birthday." I am very glad my birthday is not May 15th, as I infinitely prefer the verses of May 12th to those of May 15th. F. N.

The appointment of Miss Stevenson to the matronship of the 3rd Northern General Hospital recalls the story of the bombardment of the Hartlepools Hospital, for it will be remembered that Miss Stevenson was matron there at that time. Miss Stevenson actually saw the first shots fired from the German boats, and though too busy attending to casualties, for which emergency beds had to be placed in every available space, including the corridors, to watch the bombardment, was unpleasantly aware of the course of events, as the hospital itself was struck and the Nurses' Home badly damaged by German shells. The nursing staff luckily escaped injury.

Apparently the military hospitals in Holland are greatly in need of reorganisation. One always associates spotless cleanliness, order, and method with the Dutch people, but if report speak true these virtues are not very noticeable in the army hospitals of the Netherlands. The buildings are said to be unsuitable and insanitary; the equipment of hospital dressings, medical and surgical stores, inadequate; the staff of old soldiers and attendants quite insufficient and unsuited even to do the domestic work, while the actual nursing is done by soldiers who have had no nursing training. There are not wanting in Holland well-trained and able women capable of organising an efficient Nursing Service, of training nursing orderlies, and dealing with the varied evils from which the hospitals now suffer, which necessarily cause great loss of life amongst the soldiers of any country which permits such a state of things to exist. We hope the Dutch Government will place the nursing arrangements in the hands of an organised Nursing Service drawn from the most highly educated women nurses in the country, giving a matron-in-chief full and ample powers completely to reorganise the nursing arrangements in all military hospitals.

